# Membrane trafficking in neuronal maintenance and degeneration

**DOI:** 10.1007/s00018-012-1201-4

**Published:** 2012-11-08

**Authors:** Dong Wang, Chih-Chiang Chan, Smita Cherry, P. Robin Hiesinger

**Affiliations:** 1 Department of Physiology, University of Texas Southwestern Medical Center, Dallas, TX 75390-9040 USA; 2 Green Center for Systems Biology, University of Texas Southwestern Medical Center, Dallas, TX 75390-9040 USA; 3 Present Address: Institute of Physiology, National Taiwan University, Taipei, Taiwan

**Keywords:** Autophagy, Endosome, Lysosome, Huntington, Alzheimer, Parkinson

## Abstract

Defects in membrane trafficking and degradation are hallmarks of most, and maybe all, neurodegenerative disorders. Such defects typically result in the accumulation of undegraded proteins due to aberrant endosomal sorting, lysosomal degradation, or autophagy. The genetic or environmental cause of a specific disease may directly affect these membrane trafficking processes. Alternatively, changes in intracellular sorting and degradation can occur as cellular responses of degenerating neurons to unrelated primary defects such as insoluble protein aggregates or other neurotoxic insults. Importantly, altered membrane trafficking may contribute to the pathogenesis or indeed protect the neuron. The observation of dramatic changes to membrane trafficking thus comes with the challenging need to distinguish pathological from protective alterations. Here, we will review our current knowledge about the protective and destructive roles of membrane trafficking in neuronal maintenance and degeneration. In particular, we will first focus on the question of what type of membrane trafficking keeps healthy neurons alive in the first place. Next, we will discuss what alterations of membrane trafficking are known to occur in Alzheimer’s disease and other tauopathies, Parkinson’s disease, polyQ diseases, peripheral neuropathies, and lysosomal storage disorders. Combining the maintenance and degeneration viewpoints may yield insight into how to distinguish when membrane trafficking functions protectively or contributes to degeneration.

## Membrane trafficking and maintenance: what keeps the healthy neuron alive?

**Fig. 1:**
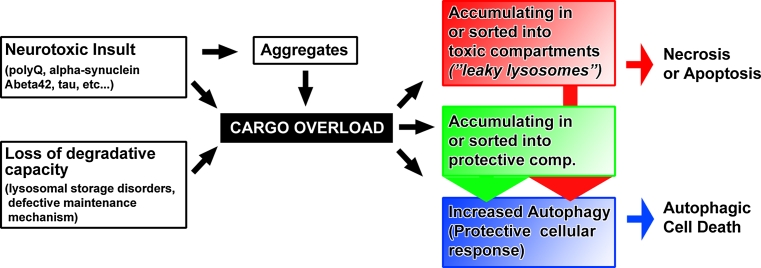
Aberrant membrane trafficking can be the cause or effect of intracellular cargo overload. Accumulation of different disease proteins can lead to a ‘cargo overload’ phenotype in a manner similar to loss of normal degradative capacity and maintenance. Aberrant cargo can be the cause of membrane trafficking defects by accumulating in toxic compartments (*red*) or protective compartments (*green*). In addition, some neurotoxic cargoes have been suggested to cause toxicity as soluble proteins (e.g., polyQ) or are known to be secreted and ultimately lead to extracellular accumulations (e.g., Abeta42). In all cases, the trafficking of neurotoxic cargoes in the cell may trigger autophagy as a protective cellular response that itself can contribute to the pathology by causing cell death

Neurons are extraordinarily polarized cells. Both axonal and dendritic branches represent challenges especially with regard to membrane trafficking for both neuronal function and maintenance. Synaptic vesicles outnumber any other membrane compartment on the presynaptic site. Even though neurotransmitter release is an extensively studied process, we still understand little about the generation, sorting, and maintenance of synaptic vesicles [1]. In particular, it is not clear how dysfunctional vesicle proteins or complete vesicles are recognized, sorted, and degraded. In addition, we know a lot about specialized membrane trafficking on the postsynaptic site, especially from pioneering studies on the recycling of neurotransmitter receptors [2, 3], and yet, similar to the presynaptic site, the sorting, quality control, and degradation of the underlying trafficking compartments are largely uncharacterized. Failure to provide adequate quality control and degradation of pre- or post-synaptic trafficking compartments leads to the accumulation of dysfunctional intracellular machinery [4, 5]. Accumulations of dysfunctional membrane compartments may be toxic to the neuron, for example if acidified compartments start leaking protons or activated proteases. Alternatively, the neuron may execute a cellular reaction to aberrant accumulations that may itself become the cause of synaptic dysfunction, degradation, or cell death. Hence, a decrease in normal degradative capacity may lead to degeneration either because of inherent toxicity of accumulating cargo or because of a toxic cellular reaction to such accumulations (Fig. 1). Importantly, both the accumulating compartments and the cellular response may also, at least initially, serve protective roles. Hence, neither the observation of aberrant intracellular compartments nor a cellular clearance response can straightforwardly be interpreted as either toxic or protective. As we will see below, the same considerations need to be discussed in the context of defective normal maintenance and neurotoxic protein accumulations associated with neurodegenerative diseases (Fig. 1). We therefore think that the study of machinery employed by wild-type neurons to maintain healthy synapses is likely to reveal fundamental insights into common features of neurodegenerative disorders that are characterized by intracellular accumulations. Towards this goal, we will first briefly highlight the neuronal maintenance roles of the three known endomembrane sorting and degradation mechanisms: autophagy, ubiquitous endolysosomal degradation and neuron-specific ‘sort-and-degrade’ [4].

### Autophagy

Autophagy is a conserved intracellular degradation pathway that clears proteins and organelles from the cytoplasm and makes resources available to the cell in response to starvation. Autophagy is classified into three major types: chaperone-mediated autophagy (CMA), microautophagy, and macroautophagy [6]. Macroautophagy mediates bulk degradation of cytoplasmic components including organelles [7]. The importance of membrane trafficking machinery that keeps neurons healthy is highlighted by seminal studies on the role of macroautophagy (hereafter referred to as autophagy) in adult neurons. Loss of autophagy in neurons of otherwise wild-type mice (through loss of *atg5* or *atg7*) leads to adult-onset degeneration [8, 9]. These finding suggests that low levels of autophagy, even though not readily detectable by microscopy in wild-type neurons, are required for neuronal maintenance in healthy neurons [10]. Neurons are long-lived postmitotic cells, in which dysfunctional proteins and damaged organelles cannot be transferred to daughter cells, which is often argued to make them more sensitive to accumulation of undegraded cargo [11]. In addition to the maintenance role, autophagy is a known cellular response to intracellular cargo overload. As such, autophagy can be considered a salvage mechanism to remove excess intracellular debris. The degradation of aggregated proteins in the cytosol by autophagy is well characterized [12, 13]. At low levels, increased autophagy acts protectively. Mild induction of autophagy can confer partial neuroprotection [14, 15]. However, the salvage mechanism has an emergency exit: increased autophagy above a certain threshold is a cell death mechanism. Hence, the same autophagic cellular reaction can function protectively and turn into a cell death mechanism in response to increasing cargo load [7, 16, 17]. Consequently, upregulation of the autophagic maintenance mechanism may not be a generally applicable solution to combat cargo overload in neuronal health and disease (Fig. 1) [18–20].

### Ubiquitous endolysosomal degradation

**Fig. 2:**
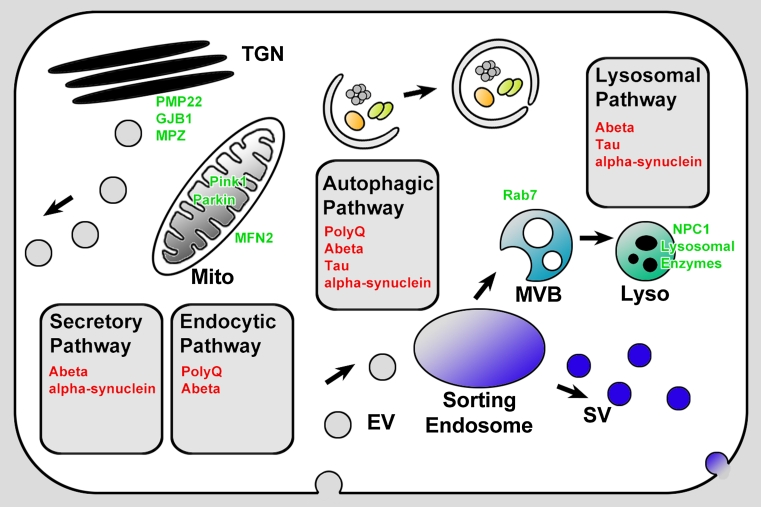
Neurodegeneration disease proteins and membrane trafficking. Neurotoxic proteins are shown in *red* in the membrane trafficking pathways where they are known to accumulate: secretory, endocytic, autophagic, or lysosomal pathways. Proteins encoded by disease genes that directly affect membrane trafficking and that are discussed in this review are shown in *green*. *TGN* trans golgi network, *Mito* mitochondrium, *EV* endosomal vesicle, *MVB* multivesicular body, *SV* synaptic vesicle, *Lyso* lysosome

Endocytosed material traffics through transport vesicles to early endosomes which mature into multivesicular bodies (MVBs) or late endosomes and finally fuse with ER/Golgi-derived vesicles that contain degradative machinery to form lysosomes (Fig. 2). Phagocytosis and autophagy provide alternative entry points for larger molecules and organelles. Late endosomes, lysosomes, and autophagosomes are the primary organelles for endomembrane degradation. Mutations in proteins that affect late endosomal or lysosomal function are often initially viable but cause intracellular membrane accumulations [21]. In many cases, these accumulations (or a cellular response to them) ultimately cause neuronal cell death. Consequently, such mutants have been utilized to model neurodegeneration and lysosomal storage disorders [22–24].

An increasing pH gradient in the endolysosomal pathway is required for intracellular trafficking [25, 26]. The maturation of endosomes into lysosomes is marked by the progressive acidification of the compartment, to ultimately allow for the activation of acidification-activated proteases in lysosomes. Impaired endocytic trafficking by disrupting the pH gradient or mutations in the cargo-carrier proteins can cause neurodegeneration [27–29]. Although endolysosomal degradation occurs ubiquitously, dysfunctional degradation firstly causes problems in tissues in which the substrate turnover is high. As discussed below, this may be a reason why two-thirds of lysosomal storage disorders affect the central nervous system and cause progressive cognitive and motor decline [21]. The accumulation of undegraded substrates can be the primary cause that exerts toxic effects on other cellular functions. Conversely, the undegraded aggregates may be the result of an independent dysfunctional membrane trafficking process (Fig. 1).

### Neuronal ‘sort-and-degrade’

Autophagy and endolysosomal degradation are ubiquitous mechanisms thought to be required for the function and maintenance of all cells. We have recently identified a neuron-specific degradation pathway [29]. Mutations in *v0a1* (*v100* in *Drosophila*) lead to neuron-specific degradation defects and are, to our knowledge, the first mutations in a neuron-specific regulator of membrane trafficking shown to cause neurodegeneration. Loss of *v100* causes intracellular sorting and degradation defects downstream of endocytosis [29, 30]. Similarly, mutations in the synaptic vesicle SNARE neuronal Synaptobrevin (*n*-*syb*) cause intracellular sorting and degradation defects that lead to slow adult-onset degeneration in *Drosophila* [31]. Both *v0a1* and *n*-*syb* are neuron-specific membrane trafficking proteins that predominantly function at synapses [4]. It is interesting to note that loss of neuronal degradative capacity in these mutants may cause a similar ‘cargo overload’ problem in neurons as the accumulation of disease proteins due to increased expression, misfolding, or aggregation (Fig. 1). In both cases, autophagy is initiated as a cellular response—with both a protective and cell death potential as discussed above. It is not clear whether the *v100*- and *n*-*syb*-dependent neuronal ‘sort-and-degrade’ mechanism has a specificity for synaptic cargo. Alternatively, *v100* and *n*-*syb* may simply increase general neuronal degradative capacity predominantly at synapses. Both *v100* and *n*-*syb* have close homologs (*v0a2*-*4* and *cellubrevin*) that exert very similar functions in other cell types.

The idea of a degradation mechanism with specificity for synaptic cargo is supported by the knowledge that synapses contain numerous specializations of membrane trafficking. Both *v100* and *n*-*syb* function on synaptic vesicles and are required for normal neurotransmitter release, suggesting a molecular link between the synaptic vesicle cycle and synaptic endolysosomal ‘sort-and-degrade’. A similar link has recently been identified in the *skywalker* mutant in *Drosophila*. *skywalker* encodes a rabGAP that functions at the intersection of synaptic vesicle recycling, sorting, and degradation [32]. The recent discovery of many novel synaptic endosomal Rab GTPases further suggests the existence of more neuronal membrane trafficking machinery required for neuronal maintenance [33, 34].

### Membrane trafficking and neurodegeneration: what kills the degenerating neuron?

In the following sections, we will discuss known membrane trafficking defects for several prominent neurodegenerative diseases. For all these diseases aberrant membrane trafficking has been observed and linked to neuronal degeneration. We will focus on the basic questions raised by our review of neuronal maintenance mechanisms. What are the causal relationships between the observed defects in membrane trafficking and pathology? When do they represent primary defects or cellular responses? In addition, we will focus on the idea of neuronal degradative capacity. How far do intracellular accumulations in the endomembrane system cause a ‘cargo overload’ situation similar to the loss of degradative maintenance mechanisms (Fig. 1)? With these questions in mind, we will review which intracellular accumulations are known in different neurodegenerative diseases, what is known about their inherent toxicity, and the cellular membrane trafficking reaction to these accumulations (Fig. 2).

### PolyQ diseases

**Fig. 3:**
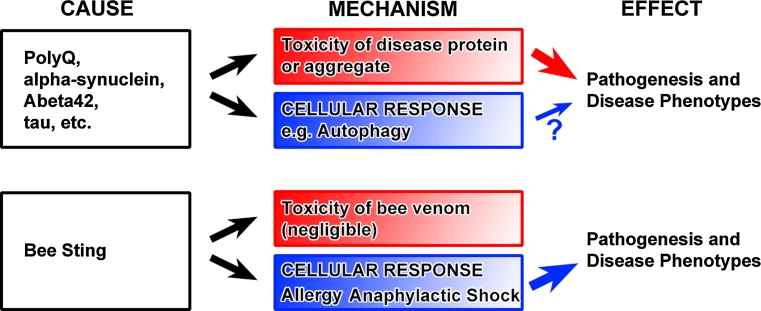
The ‘Bee Sting’ analogy. Expression of polyQ proteins, alpha-synuclein, Abeta42 or tau are sufficient to induce degeneration in neurons. Hence, they are a direct cause of pathogenesis and disease phenotypes. Properties like intracellular aggregation and inclusion in membrane compartments are easily interpreted to have intrinsic toxicity and provide a disease mechanism linking cause and effect (*red box*). Cellular responses to these proteins have been less studied. For example, aberrant autophagy is more often interpreted as impaired through toxic properties of the disease proteins than as a functional cellular response that may itself cause cell death. However, the ‘disease mechanism’ of a bee sting may remind us that not only intrinsic toxicity but also cellular responses may be a cause of pathogenesis

PolyQ disorders are caused by mutations that lead to the expansion of a polyglutamine (PolyQ) stretch in known disease proteins. These disorders include Huntington’s disease (HD), several types of spinocerebellar ataxia (SCA), and spinobulbar muscle atrophy (SBMA). The onset and severity of these disorders positively correlate to at least some extent with the length of the CAG repeat coding for glutamine [35–39]. Intracellular PolyQ protein aggregates formed by the mutant proteins are the hallmark of the diseases. In all polyQ diseases, correlations between aggregate formation and disease phenotypes are well established. The length of the polyQ stretch directly affects the propensity of the mutant protein to aggregate, which in turn correlates to some degree with the severity of disease phenotypes. Thus, the causal relationship between polyQ protein and phenotype seems deceivingly clear. In what way can the idea of polyQ as the cause of disease phenotypes be misleading? First, the correlation of the observed polyQ inclusions in different parts of patient brains do not necessarily correlate with where the degeneration occurs; in fact, some neurons that seem to evade degeneration exhibit most polyQ inclusions [40]. These and other data have been interpreted to argue that polyQ inclusions may represent protective cellular responses—a common theme across disorders characterized by undegraded protein accumulations. Second, a direct causal relationship between polyQ length, aggregation, and toxicity could still hold if the aggregates were not toxic themselves, but instead the result of a cellular response to the aggregates or pre-aggregate variants of polyQ proteins. The ubiquitin/proteasome system (UPS) and autophagy are the major clearance mechanisms and cellular responses to aberrant intracellular accumulations. In addition, a defect or overload of the UPS can trigger a further increase in autophagy [13, 41], which itself can be sufficient to cause cell death. In such a scenario, the polyQ protein remains the primary cause of a chain of event that results in degeneration, but the actual cause of pathogenesis may be the cellular response. The importance of this difference can be highlighted by a comparison to allergic reactions (see ‘bee sting analogy’, Fig. 3). An allergen can be the cause of severe pathology and even cell death in a dosage-dependent manner; however, the actual disease mechanism is an (over-) reaction of a cellular response. Reducing this cellular (and normally protective) response is where therapeutic intervention is required and successful. This analogy of course cannot explain polyQ-induced pathology in its entirety. However, it is currently unclear to what extent aberrant protein aggregates and the inclusion formation through endomembrane system responses cause degeneration (Fig. 3). This is not only the case for polyQ aggregates, but several intracellular cargos that are considered neurotoxic based on their occurence in the diseases discussed in this review. We will highlight commonalities of polyQ diseases mainly based on examples from polyQ-Huntingtin and polyQ-Sca1. Specifics on the different polyQ diseases are reviewed in detail elsewhere [36, 42, 43].

PolyQ proteins have been observed to accumulate in both the nucleus and cytoplasm where they directly or indirectly affect a plethora of cellular functions. Potential primary effects of PolyQ proteins include disrupted transcription, mitochondrial function, Ca^2+^ homeostasis, axonal transport, and the UPS and autophagosomal clearance systems [38]. Membrane trafficking is directly or indirectly affected in every single one of these potential targets of polyQ toxicity, which may be best studied for Huntingtin-polyQ. HD is the most common form of polyQ disorders and is caused by mutant variants of the *huntingtin* (*htt*) gene with elongated glutamine repeats over 36–40 residues [44]. Wild-type *htt* protein is ubiquitous and mostly cytosolic [36]. An N-terminal fragment containing the polyQ stretch is produced by the proteolysis of mutant *htt*, associates with membranes, and may be sufficient to cause pathology [45]. Loss of *htt* in knock-out mice leads to embryonic lethality [46–48]. A polyQ-extended variant of *htt* can rescue the embryonic lethality, suggesting that the wild-type function and polyQ disease mechanism may be different [49]. Htt-polyQ overexpression has been shown to both increase synaptic function at the mouse neuromuscular junction [50] and reduce synaptic function at the *Drosophila* larval neuromuscular junction [51]. Synaptic defects can be partially rescued by overexpression of Rab11, a recycling endosome protein, in *Drosophila* [51, 52]. Taken together, extensive work on Htt-polyQ indicates a pathological role of the polyQ oligopeptide with commonalities in all polyQ diseases. However, it is difficult to pinpoint a direct effect of polyQ on membrane trafficking and many of the observed changes are likely the indirect results of multiple changes of the cellular physiology. In addition, RNA-based toxicity of the CAG repeat containing mRNA encoding the polyQ stretch has been shown in *Drosophila* and is discussed elsewhere [53, 54].

Both the UPS or autophagic clearance systems can be triggered by intracellular accumulations in several polyQ diseases, including SBMA [55], HD [12], and Sca1 [13]. Upregulation of chaperones like Hsp70 are common in polyQ diseases [56–59]. It is less clear how far the UPS or autophagy are negatively affected by polyQ proteins prior to or after aggregation. Although inclusions in HD include ubiquitinated proteins and proteasome components, the UPS is not significantly impaired in mouse models for HD or Sca7 [60, 61]. In contrast, macroautophagy may be directly impaired by Htt-polyQ [11, 62]. Pharmacological upregulation of macroautophagy has been shown to be effective in reducing neuronal aggregates and slowing the progression of neurological symptoms in fly and mouse models of HD [63]. However, in some cases, defective autophagy is deduced from the observation of massively increased autophagy, including intermediates of autophagosome maturation. We have recently observed such an increased autophagy phenotype in *Drosophila* neurons mutant for a neuron-specific endomembrane degradation mechanism [4, 31]. These neurons clearly exhibit endomembrane trafficking, intracellular degradation defects, and dramatically increased autophagy with many autophagosome intermediates that are not normally observed. However, measurement of autophagosome acidification and activity of autophagy proteases (Cathepsins) revealed that autophagy is functional in these neurons at least to a very late step [31]. This finding may serve as a cautionary note that the observation of aberrant autophagy may not necessarily be the result of a defect in autophagy but can also result from a dramatic upregulation of fully functional autophagy as a cellular response to a neurotoxic insult. As discussed above, this cellular response may initially be protective but can become a cell death mechanism as a function of levels. This common theme is observed in all neurodegenerative diseases discussed here, including Alzheimer’s disease and other tauopathies, Parkinson’s disease, peripheral neuropathies and lysosomal storage disorders.

### Alzheimer’s disease

Alzheimer’s disease (AD) is the most common neurodegenerative disorder. The cause (or causes) of AD remain enigmatic and no cure is available. More than 90 % of AD are sporadic, occur in patients above the age of 65, and are not caused by known individual disease mutations. Less than 10 % of AD cases have an earlier onset and half of these are categorized as familial Alzheimer’s disease (FAD) caused by known disease mutations in one of at least three genes: amyloid precursor protein (APP), Presenilin 1, or Presenilin 2. All three genes are directly linked to the generation of amyloid β (Aβ) peptides [64, 65]. The deposition of extracellular Aβ protein in characteristic plaques is a defining hallmark of AD. The plaques consist of 40–42 amino acid Aβ polypeptides, which are the proteolytic cleavage products of the larger APP by β-and γ-secretase activity. The 42 amino acid variant is the predominant neurotoxic polypeptide and the disease-causing agent according to the predominant ‘amyloid hypothesis’. However, despite a wealth of seminal studies on the generation, trafficking, and toxicity of Aβ, it is still surprisingly unclear how intracellular and extracellular Aβ are causally linked to AD pathology, as comprehensively discussed elsewhere [66–71].

The role of membrane trafficking in AD pathology has increasingly been appreciated since the realization that extracellular Aβ accumulations do not necessarily correlate with disease phenotypes. Hence, intracellular Aβ processing and trafficking may have direct or indirect neurotoxic effects. APP is a type I transmembrane protein. γ-Secretase can function presumably both on the plasma membrane (leading to extracellular Aβ) and on endosomal compartments (leading to cytoplasmic Aβ). The correct sorting of all players [APP, β- and γ-secretase, and β-site APP-cleaving enzyme 1 (BACE1)] to the early endosomes is a prerequisite for this process [68, 72]. Increased Aβ production leads to Aβ accumulation in endosomal compartments [73]. Furthermore, dysfunction of retromer proteins has been shown to enhance AD pathology through blocked trafficking of APP and BACE1 from the early endosome to the trans Golgi network (TGN) [74–77]. Finally, failure to release Aβ into the extracellular space through exosome secretion has been discussed as a mechanism that leads to directly or indirectly neurotoxic Aβ accumulations in the endosomal system [78]. Are any of these endosomal defects causative for AD pathology? Importantly, enlarged and dysfunctional endosomes have been observed as an early sign for AD [79, 80]. Hence, intracellular changes caused by increased Aβ in the endosomal system may indeed be at least part of the cause of AD pathology. However, these observations do not show whether the intracellular Aβ accumulations are themselves toxic per se or whether it is the cellular reaction to these accumulations that kills the neuron. With this concern in mind, autophagy is again a prime suspect for a secondary cellular response that may kill the cell. However, autophagy has been implicated at many steps, including the generation of Aβ [81], making it difficult to pinpoint the role of autophagy as cause or effect. In addition, induction of autophagy has also been shown to promote clearance of Aβ in neuronal culture [82]. However, the de-correlation of Aβ accumulation and pathogenesis makes this result more difficult to interpret. Is autophagy at least partially causative or a cellular response in AD disease neurons? Mislocalization of the v-ATPase component V0a1 was recently proposed to be at least partially responsible for causing degenerative phenotypes in Presenilin 1 (PS1) knock-out cells by rendering autophagosomal and lysosomal compartments dysfunctional [27]. These findings provide a potential link to the neuronal maintenance function of V0a1 (V100) described in *Drosophila* [29, 83]. However, closer investigation of PS1 and PS2 double mutants revealed that lysosomal acidification is probably not responsible for the observed defects, shedding doubt on this mechanism as a cause for AD [84–86]. Instead, PS1 and PS2 deficiency has more complicated effects on the cellular physiology that include defects in Ca^2+^ homeostasis. These findings raise more questions about cause and effect that are discussed elsewhere [86, 87]. While it is clear that autophagy can contribute to the degeneration in AD neurons, it remains unclear whether this is due to defective autophagy or to overactivation of functional autophagy. In addition, Aβ42 was found to accumulate in the multivesicular bodies and disrupts lysosomal and MVB membrane integrity [88, 89]. Furthermore, potentially toxic Aβ accumulations have been linked to ER and mitochondrial dysfunction, highlighting the difficulties in pinpointing the precise timeline and causalities of intracellular defects [90–93].

To what extent are endomembrane degradation and neuronal degradative capacity in general responsible for the slow, progressive accumulation of undegraded Aβ and possibly pathogenesis? A study using metabolic labeling in the central nervous system of patients with the most common late-onset form of AD revealed that production of Aβ40 and Aβ42 were normal, but their clearance impaired [94]. This important finding highlights the idea of neuronal degradative capacity as a maintenance mechanism, as discussed above. Both endolysosomal degradation and autophagy are likely endomembrane degradation mechanisms that are at least partially responsible for clearance.

Axonal dystrophy is another hallmark of AD neurons [28, 95]. Dystrophic neurites are characterized by axonal swellings that contain undergraded intracellular cargo, including autophagosomes [96]. Lysosomal and autophagosomal defects have been shown to cause dystrophic swellings reminiscent of similar defects in AD mouse models [28]. Many accumulations in axonal swellings of AD patients and mouse models have been shown to contain Reticulon 3 (RTN3), a negative regulator of BACE1. Interestingly, overexpression of RTN3 is sufficient to cause dystrophic neurites [97]. In addition, inhibition of RTN3 aggregation was shown to reduce amyloid deposition and the formation of dystrophic neurites in an AD mouse model [98]. Finally, RTN3 has been suggested to directly affect autophagic clearance [99]. RTN3 may thus directly contribute to AD pathology by affecting membrane trafficking [100].

In addition to Aβ plaques and axonal dystrophy, another key morphological hallmark of AD are neurofibrillary tau tangles. Intracellular tau accumulations pose the same principle questions discussed above: to what extend are the accumulations themselves toxic or cause a toxic cellular response? The effect of tau tangles will be discussed in more detail in the next section.

### Tauopathies and FTDP-17

Tauopathies collectively describe neurodegenerative diseases characterized by the pathological aggregation of tau protein in neurofibrillary tangles. The list includes AD, Pick’s disease, supranuclear palsy, and other neurodegenerative diseases with otherwise divergent cause and pathogenesis [101]. The observation that tau tangles occur in many different neurodegenerative diseases raises the key question posed in this review, namely to what extent are accumulations a cause or an effect of intracellular membrane trafficking defects and degenerative pathology? In the case of tau, key insight comes from a single rare genetic neurodegenerative disorder caused by mutations in the tau gene itself (MAPT). These mutations cause frontal temporal dementia (FTD) with parkinsonism linked to chromosome-17 (FTDP-17) [102, 103]. Over 30 different tau gene mutations have been identified in families with FTDP-17 [104]. In all cases, tau forms filamentous inclusions (tangles) composed of hyperphosphorylated tau [105, 106]. These findings demonstrate that tau dysfunction is sufficient to trigger neurodegeneration and dementia without the presence of other neurotoxic insults. As in all other neurodegenerative diseases associated with tau tangles, it is less clear whether hyperphosphorylated tau, tau tangles, inclusions, or a cellular reaction cause pathology [107–110].

Tau is a microtubule-associated protein (MAP) that stabilizes microtubules [111, 112]. However, tau has numerous binding partners and localizations that include the plasma membrane, the actin network, and SH3 domain proteins, suggesting that many roles of tau still remain to be defined [113]. Hyperphosphorylated tau loses its microtubule stabilizing function, resulting in cytoskeletal changes whose contributions to pathogenesis are poorly understood [114]. Phosphorylation of tau has been proposed as an essential physiological process during development and a normal cellular response to environmental stress [115–120]. Both loss of function and toxic gain of function of tau may contribute to the progress of FTD and AD [101, 113]. Under physiological and pathological conditions, tau is subject to many different posttranslational modifications including phosphorylation, acetylation, nitration, sumoylation, and ubiquitination [101, 113]. In addition, tau tangles have been somewhat de-correlated from pathology in a tauopathy mouse model where memory improvements could be induced despite continued accumulation of tangles [107]. Another tauopathy mouse model indicated that synapse loss and microglia activation can precede the appearance of tau tangles [121]. Hence, similar to Aβ plaques in AD, the morphological hallmark of tauopathies may be an effect of an earlier cause of the disease. This notion leave open the possibilities that tau tangles further contribute to the pathology or function protectively or do both by transition from an initially protective reaction to a pathological process [101, 113, 122]. Membrane trafficking, and autophagy in particular, may be a key to understand these processes.

Interestingly, studies on tau knockout mice did not reveal overt neurodegeneration and neuronal dysfunction phenotypes [123]. However, reducing endogenous tau in an AD mouse model ameliorates pathology [124] and Aβ toxicity may at least be partially mediated by tau [125, 126]. These findings suggest that it is indeed the presence of tau (in tangles or prior) that can directly contribute to pathology. Numerous mechanisms for this contribution have been proposed. Apart from the idea that hyperphosphorylated tau may be toxic itself, aggregated tau has also been implicated in the dysfunction of mitochondria resulting in production of reactive oxygen species (ROS), which can cause cell death [127]. With respect to membrane trafficking, the key idea is again an implication at the level of (attempted) clearance: tau is thought to be degraded through the ubiquitin/proteasome and lysosomal degradation pathway. Accumulated abnormal tau triggers the ubiquitin/proteasome pathway (UPS) and ultimately autophagy [5, 128]. In AD patients, tau aggregates in the tangles are often ubiquitinated implying a defect of proteasome degradation [93]. A direct block of proteasome degradation by tau has been observed [129], which may presumably increase an autophagosomal reaction [5]. Direct implication of autophagy has been shown, and inhibition of autophagy enhances tau aggregation and cytotoxicity [130–132]. Hence, these findings support a protective role of autophagy in tauopathies. However, as we have discussed before, increased autophagy beyond a certain threshold may start to contribute to pathogenesis.

### Parkinson’s disease

Parkinson’s disease (PD) is a degenerative disorder of the central nervous system that is characterized by the degeneration of dopaminergic neurons in the midbrain leading to movement disorder [133, 134]. As in the case of AD, more than 90 % of PD cases have no known cause. Around 5 % of PD cases are familial and caused by known disease mutations in one of several genes, including alpha-synuclein (SNCA), parkin (PRKN), leucine-rich repeat kinase 2 (LRRK2 or dardarin), and PTEN-induced putative kinase 1 (PINK1) [134–136]. In addition, genome-wide association studies continue to identify additional potential susceptibility genes [137, 138], including tau [139]. The pathological hallmark of PD are Lewy bodies (LBs), abnormal intracellular protein accumulations that consist primarily of α-synuclein (α-syn). Neurodegenerative disorders with this hallmark are also called synucleopathies. α-syn is highly abundant in presynaptic nerve terminals where it localizes to both the membrane and the cytosol [140]. Mutations in α-syn or overexpression of α-syn can lead to the misfolding into β-sheets that form intracellular aggregates [141]. α-syn aggregate formation is further affected by several other processes, including oxidative stress [142], ubiquitination, and phosphorylation [143]. However, the toxicity of α-syn in the aggregates (LBs) is unclear, similar to protein aggregates and accumulations in HD, AD, and other tauopathies. Indeed, intermediate soluble α-syn oligomers (protofibrils) may be more toxic than the insoluble aggregates [133]. These protofibrils are known to increase the permeability of vesicles and to release dopamine in the cytoplasm where it can exert a toxic function [144, 145]. Both protofibrils and aggregates are also resistant to cellular degradation mechanisms such as the ubiquitin/proteasome system (UPS) and lysosomal degradation [143]. The accumulation of these undegraded or partially degraded proteins promotes even further aggregate formation leading to cellular toxicity or an autophagic cellular response or both, as discussed below.

Wild-type α-syn has been suggested to be involved in the regulation of synaptic vesicle trafficking [133, 146]. α-Syn binds to Synaptobrevin and affects SNARE complex assembly [147]. Deletion of α-syn or its overexpression at non-toxic levels affects neurotransmission [148]. α-Syn disrupts vesicle trafficking by impairing the re-clustering of synaptic vesicles and thus reducing the size of the synaptic vesicle recycling pool. Synaptic vesicle distribution and trafficking are also affected by mutations in *LRRK2* (leucine-rich repeat kinase2) [149]. Several presynaptic proteins, like NSF, Syntaxin 1A, SV2A, and Synapsin, have been proposed to interact with LRRK2 [149]. Mutations in LRRK2 have been shown to cause disease phenotypes [150] and to induce autophagy leading to neuronal death [151]. While mutations in *SNCA* and *LRRK2* cause autosomal dominant PD, mutations in Parkin, *PINK 1*, and *DJ*-*1* are autosomal recessive [146]. Mutations in Parkin, a ubiquitin ligase, lead to disrupted ubiquitination of target proteins and thereby impair the degradation of damaged proteins by the UPS [133]. Interestingly, proteasomal enzyme activities is significantly reduced in PD [136]. Parkin is also involved in the ubiquitination of synaptic proteins including Synphilin 1 [152] and EndophilinA [153]. The recruitment of Parkin for autophagic clearance of damaged proteins/organelles is PINK1-dependent [154]. PINK1, a mitochondrial serine/threonine kinase, regulates mitochondrial function and its loss leads to loss of ATP which in turn affects the mobilization of synaptic vesicles from the reserve pool [155]. Finally, mutations in ubiquitin C-terminal L1 (UCH-L1) affect the clearance of damaged proteins impaired by decreased deubiquitination of substrates [136]. Hence, mutations in several genes directly affect the balance between protein aggregation and degradation.

The UPS provides the clearest direct links between PD disease genes and intracellular degradation mechanisms. However, increased autophagy in some familial forms of PD is a likely compensatory response to UPS failure [156, 157]. Since blocking of the UPS is known to induce autophagy [13, 41], endomembrane degradation may play a common role highlighted here: an initially protective mechanism that above a certain threshold contributes to the pathogenesis. In addition, to this indirect effect of UPS defects as a trigger of autophagy, α-syn in particular has direct links to membrane trafficking through its function in the synaptic vesicle cycle [146] and direct interactions with proteins in the lysosomal system. Specifically, α-syn can undergo degradation via chaperone-mediated autophagy (CMA) and mutant α-syn can bind to a lysosomal receptor without being degraded. This pathological mechanism potentially clogs up the lysosomal system which also causes degradation problems for other proteins [156, 158]. Brain-specific loss of autophagy in conditional *atg7* null mutant mice leads to presynaptic accumulations of α-syn and LRRK2 as well as slow neurodegeneration [159]. Furthermore, both Parkin and PINK1 have been linked to autophagy of dysfunctional mitochondria (mitophagy) [160, 161]. Defective CMA has been observed for UCH-L1 and α-syn [162] and is accompanied by compensatory activation of autophagy. Interestingly, dopamine-modified α-syn can mimic the effects of α-syn mutants and block the CMA degradation pathway, making the affected neurons more vulnerable to other stressors or insults [158]. This might provide a partial explanation why dopaminergic neurons are the most severely affected neurons in PD. Impairment of CMA by mutant α-syn may also reduce the accessibility of other substrates for degradation and thereby exacerbate neurodegeneration [163].

### Peripheral neuropathies

Peripheral neuropathies comprise a large group of heterogeneous diseases characterized by the slow progressive degeneration of neurons of the peripheral nervous system, muscle atrophy, and motor and sensory impairment of the limbs. Among the hereditary motor and sensory neuropathies (HMSN), Charcot–Marie–Tooth (CMT) disease is the most common form and is associated with mutations in more than 45 genes involved in Schwann cells homeostasis and neuronal function. Type 1 (demyelinating CMT) affects the myelin sheath whereas type 2 (axonal CMT) is characterized by decreased motor action potentials caused by abnormalities in the axon. Although intracellular accumulations are not regarded as disease hallmarks, defects in intracellular trafficking are a common feature in most, and maybe all, peripheral neuropathies.

Many CMT-causing mutations lead to defects in various steps of intracellular trafficking including vesicle budding, cytoskeletal transport, protein degradation, and mitochondrial axonal transport [164]. Mutations in four genes account for over 90 % of all CMT molecular diagnoses, including peripheral myelin protein 22 (PMP22), gap junction β-1 (GJB1), myelin protein zero (MPZ), and mitofusion 2 (MFN2) [165]. The most common cause of CMT type 1 are mutations in PMP22, an integral membrane glycoprotein that is primarily expressed in Schwann cells. Mutations in PMP22 cause hypomyelination [166]. While the wild-type PMP22 protein resides in the plasma membrane, the mutant forms are not able to target to the plasma membrane and accumulate in the ER and Golgi [167]. Misfolded PMP22 may cause a ‘cargo overload’ phenotype with cellular responses discussed here for all degenerative diseases [168]. In further similarity to other diseases, the accumulations containing mutant PMP22 form ubiquitinylated cytoplasmic inclusions that have been suggested to play a cytoprotective role [169, 170].

Mutations in GJB1 also cause myelination defects. The GJB1 protein is a transmembrane protein that assembles to form gap-junction channels that facilitate the transport of ions and small molecules across the myelin sheath. Mutations in GJB1 lead to dysfunctional gap junctions that disrupt communication between Schwann cells and neurons [171]. GJB1 mutants are often retained in either the ER or Golgi, where they potentially affect intracellular trafficking and trigger cellular clearance responses [172–174]. Similar trafficking defects are observed for the major integral membrane protein of peripheral nerve myelin, MPZ. Some disease-associated mutant forms of MPZ proteins can be retained in the ER or Golgi, while other MPZ mutants can be incorporated into myelin and disrupt the myelin structure by dominant–negative interactions between mutant and wild-type MPZ [175].

The leading cause of the axonal CMT (type 2) are mutations in MFN2, a gene encoding a mitochondrial membrane protein that mediates mitochondrial fusion and the tethering of ER and mitochondrial membranes [176, 177]. Mutations in MFN2 not only lead to disruption in mitochondrial fusion but also interfere with axonal transport of mitochondria [178]. In neurons, it is particularly important for the mitochondria to be distributed far away to the axons and dendrites. This could be the reason why defects in mitochondrial fusion affect neurons prior to other cells. Both loss of MFN2 and expression of CMT-associated MFN2 mutants lead to slow movement of mitochondria along microtubules [178, 179]. In a rare form of CMT, type 2 mutations are found in the late endosomal rab GTPase *rab7* that may alter its functional properties and thereby endolysosomal degradation directly [180, 181]. Finally, the fact that neurons are post-mitotic cells with a long life span is the most commonly evoked argument to explain the particular neuronal susceptibility to mutations in ubiquitous genes that predominantly affect neurons.

### Lysosomal storage diseases

Lysosomal storage diseases (LSDs) result from the malfunction of lysosomal degradation. They comprise a class of more than 50 different, rare, inherited, and monogenic disorders caused by loss-of-function mutations in genes that encode proteins directly or indirectly linked to lysosomal function [21]. LSDs are particularly interesting in the context of this review, because they are clearly defined by a primary membrane trafficking and degradation defect. A further particularity of LSDs is that they typically affect a ubiquitous lysosomal function, yet nervous system defects are often the first and most prominent symptoms. Most LSDs do not affect early brain development or neuronal function during early developmental stages. All LSDs are progressive. Accumulation of metabolic substrates of a specific enzyme or protein typically lead to aberrant storage compartments. The defective proteins encoded by the mutant LSD genes can be categorized into three classes: lysosomal enzymes, non-enzymatic lysosomal proteins, and proteins involved in lysosomal biogenesis. Defects in any of these proteins can lead to accumulations that directly or indirectly affect numerous cellular processes from Ca^2+^ homeostasis to vesicle exocytosis [21]. In Pompe disease, for example, failure to digest glycogen leads to progressive accumulation of glycogen in the lysosomes and the cytoplasm. The accumulations results in lysosomal expansion in many tissues, and leads to cardiac failure and skeletal myopathy [182]. In Tay–Sachs disease (GM2 gangliosidosis), dysfunctional β-hexosaminidase A, a lysosomal enzyme to degrade ganglioside GM2, results in accumulation of GM2 in expanded lysosomal structures in the brain, and ultimately leads to neuronal death. However, the accumulated component is not always the substrate of the defective enzyme. For example, the late infantile form of neuronal ceroid lipofuscinosis (LINCL) is characterized by progressive and extensive neuronal death. It is caused by deficiencies in the lysosomal protease tripeptidyl peptidase I (TPP1), which has broad substrate specificity. Yet, the major constituent of the storage material is the subunit c of the mitochondrial ATP synthase [183]. Whether subunit c accumulation in LINCL is a primary effect of TPP1 mutations remains to be determined.

Although most LSDs exhibit accumulation of undigested metabolites in the lysosomes, not all storage is lysosomal. Mucopolysaccharidosis IIIB (MPS IIIB) disease is characterized by defects in α-*N*-acetylglucosaminidase, a lysosomal enzyme essential in degrading heparin sulfate. However, in the neurons of a mouse model of MPS IIIB, the altered structures for storage are the Golgi bodies, indicating that the problems may come from defects in vesicle trafficking from the Golgi to the lysosome [184]. This finding also indicates that LSD mutations can affect the intracellular trafficking of proteins upstream or downstream of lysosomes. Finally, dysfunctional proteins responsible for the biogenesis of or trafficking to lysosomes may also cause LSDs. In action myoclonus–renal failure syndrome (AMRF), the causative mutation is the lysosomal integral membrane protein type 2 (LIMP-2). LIMP-2 mediates the transport of β-glucocerebrosidase (β-Glc) from the ER to the lysosomes. Mutations in LIMP-2 not only result in reduced lysosomal β-Glc activity but also lead to accumulation of unidentified storage materials in a certain compartment in the brain, and ultimately cause progressive myoclonus epilepsy and ataxia [185]. All these cases imply ‘cargo overload’ or ‘traffic jam’ ideas during pathogenesis. Indeed, the defects in some LSDs are not attributable to abnormal catabolism of the substrates, but rather a defect in intracellular trafficking. For example, mucolipidosis type IV (MLIV) is characterized by the accumulation of enlarged endolysosomal compartments and impaired lysosomal exocytosis [186]. In Niemann–Pick type C1 (NPC1), the trafficking between late endosomes and lysosomes is disrupted and cholesterol accumulates in the membranes of these compartments [187]. Overexpression of Rab7 or Rab9 increases late endocytic trafficking and restores the lipid trafficking defects in NPC1 mutant cells [188]. Hence, dysfunctional late endosomal trafficking may be at least partially responsible for the neurodegeneration caused in some LSDs. In further similarity to other neurodegenerative diseases discussed here, the common cellular response is autophagy. However, it has also been shown for several LSDs that autophagy may be impaired at the level of autophagic vacuole maturation or autophagosomal–lysosomal fusion [189–191]. As noted above, there is a possibility that increased functional autophagy leads to a build-up of autophagosomal structures that can easily be misinterpreted as defects in autophagy. Defects in the maturation of autophagosomal structures are likely to lead to further activation of autophagy as a feedback response [192, 193].

## Conclusion

In this review, we compared the roles of membrane trafficking in maintenance mechanism and several major neurodegenerative diseases. The maintenance mechanisms considered here include autophagy, ubiquitous and neuron-specific endolysosomal degradation. The neurodegenerative disorders highlighted for their direct or indirect dysfunctions of membrane trafficking include PolyQ diseases like Huntington’s disease and spinocerebellar ataxias, Alzheimer’s disease and other tauopathies, Parkinson’s disease, sensory neuropathies and lysosomal storage disorders. Remarkably, loss of any of the maintenance mechanisms can cause cellular defects that share the following common features with every single one of these degenerative disorders:

1. Few or no early defects, but progressive deterioration.
2. Defects are more pronounced or occur exclusively in the nervous system even if ubiquitous mechanisms are affected.
3. Increased cargo (over-)load inside the cells.
4. Endomembrane degradation, most prominently autophagy, is either directly affected or a cellular response—or both.
5. It is unclear when accumulations and inclusions are protective or toxic.

We conclude that a better understanding of membrane sorting and degradation in healthy neurons may hold a key to understanding some remarkably similar features underlying numerous neurodegenerative diseases.
